# Adaptation and psychometric evaluation of the simplified Chinese version of the identification of functional ankle instability questionnaire in Chinese-speaking patients with chronic ankle instability disorders

**DOI:** 10.1186/s12891-020-03314-1

**Published:** 2020-05-25

**Authors:** Wei Wang, Jun Sheng, Yinchao Tang, Qingyun Xie, Meng Wei, Zhiqiang Li, Wei Zheng

**Affiliations:** 1 Department of Orthopedics, The General Hospital of Western Theater Command, Tianhui Road 270, Chengdu, 610000 People’s Republic of China; 2 Department of Rheumatism and Immunology, The General Hospital of Western Theater Command, Tianhui Road 270, Chengdu, 610000 People’s Republic of China

**Keywords:** IdFAI, Validation, Cross-culture translation, Psychometrics

## Abstract

**Background:**

The aims of this study were to adapt the Identification of Functional Ankle Instability (IdFAI) questionnaire into a simplified Chinese version and to assess its reliability, validity, and responsiveness in Chinese-speaking patients with chronic ankle instability (CAI) disorders.

**Methods:**

The simplified Chinese version of the IdFAI (SC-IdFAI) questionnaire was developed with a five-step procedure involving cross-cultural translation and adaptation. Three questionnaires, including the SC-IdFAI, Medical Outcomes Study Short-Form 36 (SF-36), and Foot and Ankle Ability Measure (FAAM), were administered to the recruited patients. Then, the Cronbach’s alpha value, intraclass correlation coefficient (ICC), standard error of measurement (SEM), minimal detectable change (MDC), Spearman’s correlation coefficient (*r*_s_), effect size (ES), and standardized response mean (SRM) were calculated to evaluate the reliability, validity, and responsiveness of the SC-IdFAI questionnaire.

**Results:**

A total of 131, 119, and 86 patients with CAI successfully completed the first, second, and third rounds of the questionnaires, respectively. Good or excellent internal consistency and test–retest reliability were found for the overall scale and subscales of the SC-IdFAI questionnaire. The values for the SEM (1.346) and MDC (3.73) were low, indicating that small clinical changes can be detected by the SC-IdFAI questionnaire. The correlations of the SC-IdFAI with FAAM and SF-36 were generally in agreement with the a priori hypotheses (85%, 34/40), suggesting the SC-IdFAI questionnaire has good construct validity. Moreover, the results suggest that the SC-IdFAI (ES = 1.123 and SRM = 1.554) has an acceptable level of responsiveness.

**Conclusion:**

The SC-IdFAI scale may be an effective tool, and it is responsive, reliable and valid for the assessment of Chinese patients suffering from CAI.

## Background

Chronic ankle instability (CAI) is one of the most common exercise-related injuries that occur. Previous studies have shown that as many as 40% of ankle sprains lead to CAI [1, 2]. The manifestations of CAI mainly include a sense of fear and instability on uneven ground, soreness and pain in the joint after a long walk, and restricted ankle joint movement [3, 4]. It has been estimated that more than 23,000–27,000 ankle sprains occur in the United States every day [5]. However, no reliable reports of the incidence of ankle sprains are available in China. Given the large population in China, the number of patients with CAI in China is also very high. In recent years, increasing attention has been paid to ankle sprains and CAI, as nationwide physical fitness campaigns were implemented, and there were increasing demands for a higher quality of life in China.

A series of studies on the development of patient-reported outcome measures (PROMs) have been conducted since the 1980s [6]. PROM data are collected mainly by asking patients to complete questionnaires independently. Through this method, a large amount of information about a specific patient group can be easily obtained. In addition, these data allow physicians to identify the severity of diseases in these patients and consequently develop appropriate treatment strategies [7]. PROMs are an important part of both clinical practice and research because they are efficient and inexpensive to administer and are reliable [8]. A PROM is considered either a generic scale or specific scale according to its purpose. Generic scales are mainly used to assess the gross conditions of various diseases in patients. For instance, the Medical Outcomes Study Short-Form 36 (SF-36) is one of the most commonly used generic scales. However, specific scales are applicable to a particular group of patients, such as the Western Ontario Shoulder Instability Index for shoulder instability [9], the Foot and Ankle Ability Measure (FAAM) for different neuromusculoskeletal abnormalities in the foot/ankle [10], the Ankle Instability Instrument (AII), Cumberland Ankle Instability Tool (CAIT), and Identification of Functional Ankle Instability (IdFAI) for CAI [11].

Professional organizations, such as the National Athletic Trainers’ Association, recommend the use of PROMs to determine patients’ perceptions of ankle instability for treatment-related decision-making in the management of CAI [12]. Seven PROMs had been developed to assess CAI before the IdFAI questionnaire was developed, among which the CAIT and AII have been acknowledged as the two scales with the highest sensitivity and specificity. Therefore, the International Ankle Consortium has recommended the use of both the CAIT and AII for assessing CAI [11, 13]. Then, Matthew Donahue et al. developed the IdFAI questionnaire in 2012 by merging the CAIT and AII. In general, the IdFAI questionnaire is a simple but effective tool for assessing CAI [14]. When only one scale was used for assessing CAI, the IdFAI questionnaire was reported to have the highest accuracy among all similar scales [15].

Many scales can be applied in certain countries and groups of patients. With the growing number of multicenter and multicountry studies [6], the demand for widely applicable scales is increasing; the wide use of these scales would yield an appropriate level of statistical power for randomized controlled trials [16]. When an effective, credible scale is used in people of different cultures, to minimize assessment biases arising from cultural differences, it is vital that not only is the content translated but also the psychometric properties of the scale are tested [17, 18]. The IdFAI questionnaire was originally developed in English and has been culturally adapted for four populations, including Korean, Brazilian, Persian, and Japanese people, and validated [11, 13, 19, 20]. However, a cross-cultural adaptation study of the IdFAI questionnaire has not been conducted for a Chinese version, although Chinese is the first language for 1.2 billion people [21] and one of the six official languages of the United Nations [22].

Therefore, the aims of this study were to translate and adapt the IdFAI questionnaire into a simplified Chinese version (SC-IdFAI) and to evaluate the reliability, validity, and responsiveness of the SC-IdFAI questionnaire in a cohort of native Chinese-speaking patients with CAI disorders.

## Methods

### Translation and cross-cultural adaptation

The original version of the IdFAI questionnaire was translated according to the principles reported in previous studies [6, 23]. In brief, the translation included the following several steps. (1) For the forward translation step, two native Chinese speakers highly familiar with the English language (one translator was an orthopedist in the hospital, while the other was a professional translator without a medical background) were asked to translate the IdFAI questionnaire into a simplified Chinese version. (2) For the discussion I step, all the investigators discussed the two independently translated versions, and finally, the two versions were merged into a preliminary version of the SC-IdFAI questionnaire. (3) For the backward translation step, two native English speakers independently translated the preliminary version of the SC-IdFAI questionnaire into the English language. (4) For the discussion II step, the versions were discussed by the investigators again to address any discrepancies, ambiguities, or other language expression issues in the preliminary version of the SC-IdFAI questionnaire. Then, the prefinal version of the SC-IdFAI questionnaire was obtained. (5) In the pretesting step, twenty patients with CAI were asked to complete the prefinal version of the SC-IdFAI questionnaire. The questions asked by the 20 patients during the completion of the scale were also recorded. If a CAI patient had difficulty understanding a certain item, then the item was specifically modified in the third discussion. After the above standard steps were completed, the final version of the SC-IdFAI questionnaire was obtained.

### Patients and data acquisition

The consecutive native Chinese-speaking patients with CAI who were treated in our hospital (the General Hospital of Western Theater Command) between February 2016 and March 2018 were consecutively recruited. The inclusion criteria were as follows: (1) patients aged > 18 years who were capable of independently providing consent; (2) Chinese native speakers who could independently read and complete the questionnaire; and (3) those with at least two cases of severe ankle sprains, feelings of ankle instability that have been reported on multiple occasions, chronic pain in the ankle and/or “giving way” in sports activities or daily life. The exclusion criteria were as follows: 1) previous surgeries in musculoskeletal structures or fractures in the lower extremities, 2) acute injuries to the musculoskeletal structures of the other lower limb joints in the previous three months, and (3) other chronic inflammatory diseases in the lower limbs that might affect ankle function. Patients who met all the eligibility criteria and volunteered to participate were included in the study. In addition, the number of included participants also met the sample size criteria of PROMs, as recommended by Terwee et al. [24]. In brief, the sample size for internal consistency analysis should be ≥100, and the sample sizes for floor or ceiling effect, reliability, and validity analyses should be ≥50. All the participants carefully read and signed the informed consent form. The study was approved by the Ethics Committee of the General Hospital of Western Theater Command.

On the first day of admission to the hospital, the patients were required to provide demographic information and, in a quiet meeting room, to complete 3 scales independently, including the SF-36, SC-IdFAI and FAAM. One day before the beginning of physiotherapy, that is, one week after the questionnaire was completed for the first time, the patients completed the SC-IdFAI for the second time so that the test-retest reliability of the scale could be assessed. Patients undergoing related treatment in the previous week were excluded. Finally, the patients who volunteered to undergo an 8-week physiotherapy program in the hospital were asked to complete the SC-IdFAI questionnaire for the third time so that the responsiveness could be assessed.

### Instruments

The IdFAI is a scale specifically designed to assess patients with CAI. It was developed by Matthew Donahue et al. based on the CAIT and AII [14]. It has the advantages of both the CAIT and AII scales and thus has the following specific advantages: (1) the IdFAI questionnaire contains 10 questions, which are easily understood and can be completed by patients in a very short time; and (2) the questionnaire has very high accuracy in assessing patients. The IdFAI questionnaire alone can yield higher accuracy than the CAIT or AII [15]. The IdFAI questionnaire consists of the following three subscales: (1) factor 1, concerning the patient’s history of ankle sprains (items 5, 6, 7, and 10); factor 2, concerning the presence and severity of ankle instability (items 1, 2, 3, and 4); and factor 3, concerning the patient’s functional performance in daily living and other physical activities (items 8 and 9). Because the first question is a nonchoice question, the answer is not included in the total score, while the scores of the other nine questions are summed to obtain the final score. The total score ranges from 0 to 37, with higher scores indicating poorer ankle function. Generally, if the total score is > 10, the patient is considered to have CAI [20].

The FAAM is a region-specific scale designed specifically for the assessment of foot and ankle functions [10]. The FAAM contains two subscales, namely, the activities of daily living (ADL) and sports subscales. The ADL subscale contains 21 questions, while the sports subscale contains 8 questions. The score for each question ranges from 0 to 4. Thus, the ranges of the total scores of the ADL subscale and sport subscale are 0–84 and 0–32, respectively, with higher scores indicating better functions. Although the FAAM is a region-specific rather than a disease-specific scale, previous studies have demonstrated that the FAAM has high validity when used in patients with CAI [25]. The SF-36 is a versatile scale for assessing quality of life, and it contains 35 questions in 8 subscales. The first four subscales can be categorized as “physical subscales,” which mainly assess the physiological functions of patients, while the remaining four subscales can be categorized as “mental subscales,” as they mainly assess the mental status of patients. Each subscale of the SF-36 scale has a specific scoring method, while the final score is converted into a centesimal score. A higher score on the SF-36 scale indicates a better mental status and physical function [26]. The abovementioned two scales have been translated into Chinese, and it has been proven that these versions have excellent responsiveness, reliability and validity [27, 28].

### Psychometric assessments and statistical analysis

The validity, reliability, and responsiveness of the SC-IdFAI questionnaire were assessed to evaluate whether the questionnaire can be applied in native Chinese-speaking patients with CAI.

The reliability tests of SC-IdFAI chiefly address measurement error, internal consistency and test-retest reliability. The degree of internal consistency is described to be the degree of interaction among scale questions [29], which is chiefly assessed by the Cronbach’s α value of the scale. When α > 0.9, 0.8, and 0.7, the scale has acceptable, good, and excellent internal consistency [10]. However, high Cronbach’s *α* values do not always indicate good validity. For instance, Cronbach’s *α* values > 0.95 generally indicate question redundancy in the scale [30]. In addition, the Cronbach’s *α* value was also calculated after the questions in the SC-IdFAI questionnaire were omitted one by one to assess the influence of each question on the *α* value, which is also a method used to assess internal consistency [24, 31]. To assess the test–retest reliability of the SC-IdFAI questionnaire, the patients were asked to answer the questions twice, with an interval of 1 week, and the answers were compared. The intraclass correlation coefficient (ICC), which was calculated for two-way analysis of the variance with a random effects model, was the assessment indicator used for the test-retest reliability, and an ICC of > 0.9 and 0.8 suggested that the scale had excellent and good reliability, respectively [32]. Measurement errors include random and systematic errors, not the patient score, and is not indicative of the real changes in the scale to be tested [33]. The error was calculated in accordance with the formula and analyzed using the standard error of measurement (SEM): SD × √ (1 – ICC). In the first evaluation, the standard deviation of all patients was expressed as SD [34]. The minimal detectable change (MDC) reflected the minimal individual change in the score that could be interpreted as a real change. It was calculated as SEM × 1.96 × √2 at an individual level and SEM × 1.96 × √2/√*n* at the group level [34]. To determine the systematic errors between the first two surveys, we generated the Bland-Altman diagram [35].

We can evaluate the validity of the CAIT-C by its construct validity and content validity. The relevance and comprehensiveness of the questions were evaluated to assess content validity [36]. The three indexes of the question comprehensiveness evaluation are patients’ feedback, the response rate, and ceiling/floor effects. Assuming that the ceiling/floor effects are lower than 15%, the response rate of the scale is more than 95%, and the patients who completed the questionnaire had no difficulty in understanding the questions, then the scale being assess has excellent comprehensiveness [24, 37]. In addition, we invited one rehabilitation specialist as well as two orthopedic specialists to help determine whether the items were relevant for the construct to be measured and for patients with CAI [36]. Since a gold standard for the assessment of SC-IdFAI criterion validity does not exist, the hypothesis test was used to assess the construct validity of SC-IdFAI. Construct validity is the extent to which the scores on a scale are consistent with hypotheses based on the assumption that the scale validly measures a specific construct [30]. In this study, FAAM and SF-36 were selected as the control scales for SC-IdFAI. Most of the questions included in the SC-IdFAI questionnaire addressed the physical conditions but not mental conditions of patients with CAI. Therefore, it was hypothesized that the results of the SC-IdFAI questionnaire highly correlated with those of the physical subscales of the SF-36 (physical functioning, role physical, bodily pain, and general health), as well as those of the FAAM, but poorly correlated with the results of the metal subscales of the SF-36 (vitality, social functioning, role emotional, and mental health). In addition, the FAAM is a region-specific scale specifically designed for patients with ankle injuries, while the SF-36 is a generic scale with wide applicability. Therefore, although the FAAM is not a disease-specific scale that was designed specifically for patients with CAI, the contents of the FAAM should be more similar to those of the IdFAI than to those of the SF-36. Thus, it was further hypothesized that the correlation between the SC-IdFAI and FAAM should be stronger than those with the subscales of the SF-36. More details on the hypotheses for the scales are shown in Table 4. Based on these hypotheses, the Spearman’s correlation coefficients (*r*_s_) of the SC-IdFAI with the SF-36 and FAAM were analyzed using the first responses from the patients for the scales. Then, the construct validity of the SC-IdFAI questionnaire was assessed on the basis of the consistency between the data and the hypotheses. Good construct validity was considered to exist when at least 75% (30/40 or more) of the predetermined a priori hypotheses were met [33]. The correlations were determined to be excellent (*r*_s_ = 0.8–1.0), good (*r*_s_ = 0.6–0.8), moderate (*r*_s_ = 0.4–0.6), fair (*r*_s_ = 0.2–0.4) or poor (*r*_s_ = 0–0.2) [38].

Responsiveness is a metric used to determine the capability of a parameter to be measured over time [36]. In this study, the responsiveness of the SC-IdFAI was evaluated by comparing the scale results before (the first time the questionnaire was completed) and 9 weeks after physiotherapeutics were administered (the third time the questionnaire was completed). The physical therapy programs included strength training (isokinetic muscle strength training and home strength training) [39, 40], balance training (star excursion balance training; SEBT) [41], and proprioceptive training (multitask training) [42].

The two indicators of responsiveness are the standardized response mean (SRM) and effect size (ES). We calculated the SRM by dividing the average change between each time point by the SD of this change. The ES was calculated as the average change in the treatment outcome within the 9 weeks before and after the physiotherapeutics divided by the SD of the SC-IdFAI score before treatment [43]. When the SRM and ES values exceeded 0.80, the effect size was considered large; when the values were between 0.51 and 0.80, the effect size was considered medium; and when the values were less than 0.50, the effect size was considered small [44].

Statistical Package for the Social Sciences, version 20.0 (SPSS, Chicago, IL, USA), was used to perform the statistical analyses. The mean value is expressed with the standard deviation (SD). The ICC values are reported with the 95%confidence intervals (CIs). A *P* value less than or equal to 0.05 is considered statistically significant.

## Results

### Patients

A total of 161 patients with CAI (including 104 males and 57 females) who were treated in our hospital between February 2016 and March 2018 and met the eligibility criteria were recruited. Of these patients, 132 (82.0% of those invited, including 86 males and 46 females) volunteered to participate in this study. All these patients completed the first survey using the scales. One week later, 119 patients (including 81 males and 38 females) completed the SC-IdFAI questionnaire for the second time during re-examination in the hospital or upon being contacted by telephone or e-mail. Among the 13 patients who did not complete the second SC-IdFAI questionnaire, 9 were excluded for receiving related treatments 1 week before (using analgesics or unclear physiotherapy), while the other 4 could not be contacted. In addition, 86 of the patients (including 62 males and 24 females) received regular physiotherapy in our hospital and completed the the SC-IdFAI questionnaire for the third time after all the treatments were completed (8 weeks later). Therefore, 132 patients were included in the analysis of internal consistency, measurement error, and validity of the SC-IdFAI questionnaire, 119 patients were included in the analysis for the test–retest reliability, and 86 patients were included in the analysis for the responsiveness of the SC-IdFAI questionnaire. The demographics of the patients who initially participated in the study are shown in detail in Table 1.

**Table 1:** Demographic and clinical characteristics of participants

Characteristics	Number (%) or Mean ± SD
Age (years)	26.5 ± 5.7
Range	18–47
Age groups	
≦20	23 (17.4%)
21–30	81 (61.4%)
31–40	25 (18.9%)
≧41	3 (2.3%)
Gender	
female	46 (34.8%)
male	86 (65.2%)
Affected side	
right	98 (74.2%)
left	34 (25.8%)
bilateral	
BMI (Kg/m^2^)	23.4 ± 4.9

*BMI* body mass index

### Translation and cross-cultural adaptation process

The forward translation, backward translation, and cross-cultural adaptation steps for the IdFAI questionnaire were all conducted as planned. Because the SC-IdFAI questionnaire was concise and easy to understand, the content of the questionnaire was not modified substantially. Twenty patients with CAI (including 10 males and 10 females) completed the prefinal version of the SC-IdFAI questionnaire in the pretesting phase, with no feedback regarding difficulty in understanding or misunderstanding the questionnaire.

Much attention was paid to the translation of the phrase “giving way,” as the understanding of this phrase was critical for the patients to complete the scale correctly. Therefore, this phrase was explained in detail in a conspicuous position in the beginning of the original version of the scale. However, no official Chinese word for “giving way” exists. After the explanations of this phrase in the original scale were carefully read, a colloquial word in Chinese (Da ruan tui) was used to reflect the meaning of this phrase. To prevent some patients from responding to the questions incorrectly due to a misunderstanding this colloquial word, this word was explained using the official Chinese language at the beginning of the questionnaire in a clearly visible location. No patients reported any content that could not be understood in the subsequent pretesting or formal survey.

### Reliability

The overall Cronbach’s *α* value of the SC-IdFAI questionnaire was 0.902, suggesting that the scale had excellent internal consistency. The Cronbach’s *α* values of the three subscales of the SC-IdFAI questionnaire were 0.897, 0.808, and 0.721, respectively, suggesting that the subscales had acceptable or good internal consistency. In addition, Table 2 shows the Cronbach’s *α* values of the SC-IdFAI questionnaire after the questions were omitted one by one, as well as the correlation coefficients between the questions and the corresponding scores of the SC-IdFAI questionnaire. No evident increase in the Cronbach’s *α* value of the SC-IdFAI questionnaire was identified after the questions were omitted one by one.

**Table 2:** The internal consistency of SC-IdFAI

Items	Corrected Item: total correlation ^a^	Cronbach’s α ^b^
Overall scale	1.000	0.902
Factor 1	0.882	0.897
*Item 5*	0.780	0.885
*Item 6*	0.815	0.880
*Item 7*	0.743	0.891
*Item 10*	0.782	0.884
Factor 2	0.725	0.808
*Item 2*	0.605	0.899
*Item 3*	0.585	0.904
*Item 4*	0.678	0.892
Factor 3	0.813	0.721
*Item 8*	0.690	0.894
*Item 9*	0.759	0.887

^a^ Calculated by the Spearman’s correlation coefficient of the subscales or items with total score

^b^ The Cronbach’s α of each item is calculated the alpha value of overall scale if this item was deleted

*SC-IdFAI* Simplified Chinese version of the Identification of Functional Ankle Instability questionnaire

**Fig. 1:**
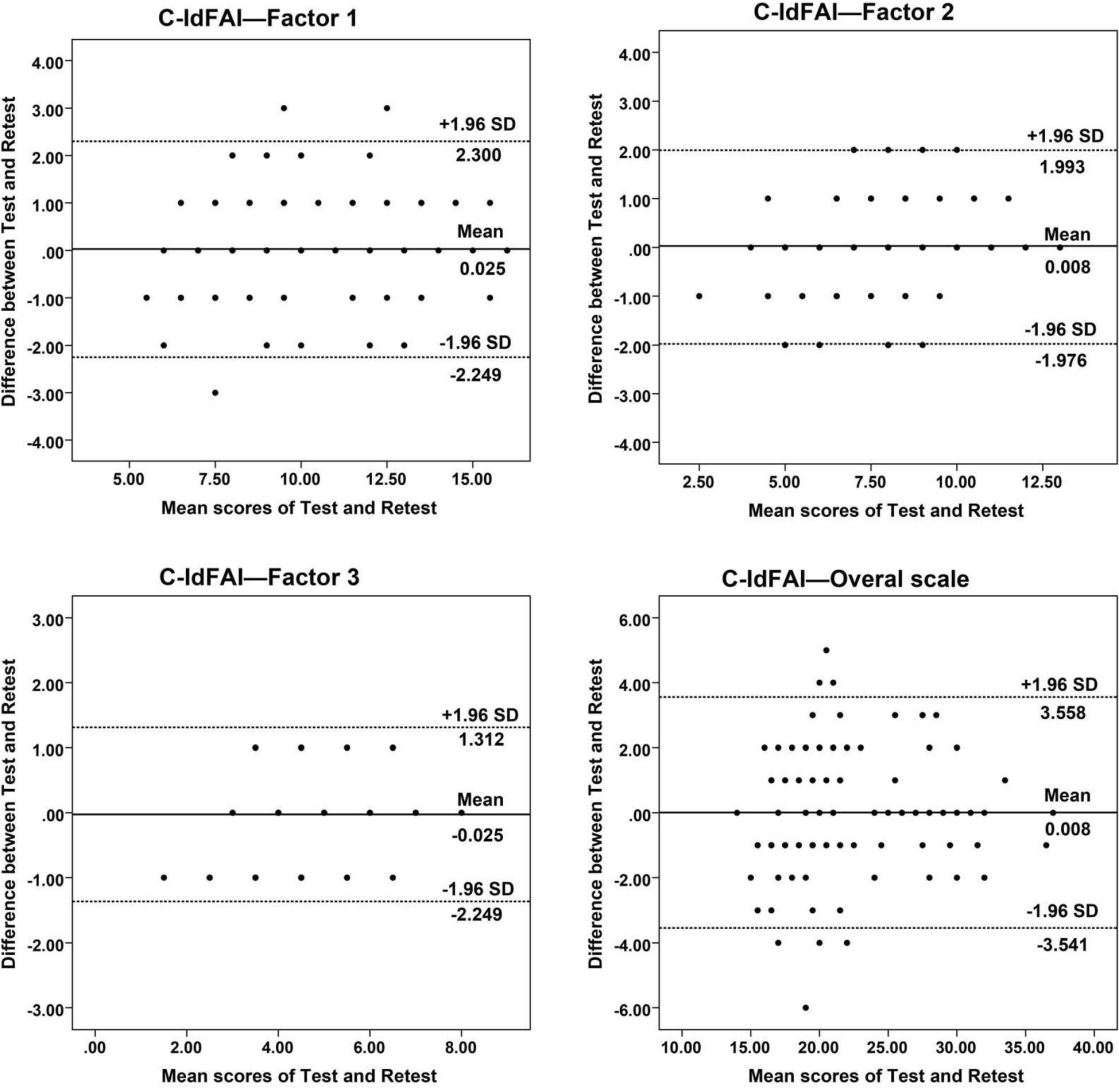
These are Bland-Altman plots of test-retest reliability of the subscales and overall scale of the SC-IdFAI. Each data point indicates how the difference between the two test sessions for an individual patient compares to the mean of the two sessions for scores of each SC-IdFAI. The interval of two sessions was 1 week. The dashed line shows the 95% (± 1.96 SD) limits of agreement

The overall ICC value of the SC-IdFAI questionnaire was 0.936, suggesting that the scale had excellent test–retest reliability. The ICC values of the three subscales of the SC-IdFAI questionnaire were 0.907, 0.881, and 0.876, respectively, suggesting that the subscales had good or excellent test-retest reliability (Table 3). In addition, the Bland–Altman plots also showed that in the first two surveys, the SC-IdFAI questionnaire and the three subscales had no evident systematic errors (Fig. 1), indicating that the SC-IdFAI questionnaire had good test–retest agreement.

**Table 3:** The floor/ceiling effects, test-retest reliability, measurement error and responsiveness of SC-IdFAI

	Floor effect ^a^	Ceiling effect ^a^	ICC (CI range)	SEM	MDC (I) ^b^	MDC (G) ^c^	ES	SRM
Overall scale	0%	1.52%	0.936 (0.909–0.955)	1.346	3.73	0.32	−1.123	−1.554
Factor 1	0%	2.27%	0.907 (0.869–0.935)	0.886	2.46	0.21	− 0.531	− 0.683
Factor 2	0%	1.52%	0.881 (0.833–0.916)	0.668	1.85	0.16	−1.221	−1.347
Factor 3	0%	2.27%	0.876 (0.827–0.912)	0.466	1.29	0.11	−1.472	−1.100

^a^ Percentage of patients with the worst (floor effect) and the best (ceiling effect) score

^b^ The MDC value at an individual level;

^c^ The MDC value at the group level

*SC-IdFAI* Simplified Chinese version of the Identification of Functional Ankle Instability questionnaire; *ICC* Intraclass correlation coefficient; *CI* Confidence interval; *SEM* Standard error of measurement; *MDC* Minimal detectable change; *ES* Effect size; *SRM* Standardized response mean

The SEM value of SC-IdFAI was 1.346. Therefore, the MDC reflecting the minimal individual and group (in this study) changes in the score that could be interpreted as a real change were 3.73 and 0.32, respectively. The SEM and MDC of the three subscales are also displayed in Table 3.

### Validity

No missing questions were found in the recovered questionnaires of SC-IdFAI in the formal survey. The mean score and SD of the overall SC-IdFAI was 24.57 ± 7.83. No ceiling effect (1.52–2.27%) or floor effect (0%) was found for the overall scale or the three subscales of the SC-IdFAI (Table 3). In addition, no patients reported difficulties or misunderstandings about the contents while completing the SC-IdFAI questionnaire. All the rehabilitation medicine and osteology experts acknowledged that the information obtained by the questions was sufficient to assess the health-related quality of life in patients with CAI, and thus, no questions were suggested to be omitted or added. These findings demonstrated that the SC-IdFAI questionnaire had good content validity.

Table 4 shows the data for the assessment of the construct validity of the SC-IdFAI questionnaire. The correlations of the SC-IdFAI questionnaire with both subscales of the FAAM were moderate or good (*r*_s_ = 0.575–0.776). In addition, the correlations of the SC-IdFAI questionnaire with the physical subscales of the SF-36 were all poor or moderate (*r*_s_ = 0.295–0.551), while the correlations with the mental subscales of the SF-36 were poor, fair, or moderate (*r*_s_ = 0.035–0.404). These findings were generally in agreement with the a priori hypotheses (85%, 34/40), suggesting that the SC-IdFAI questionnaire had good construct validity.

**Table 4:** Construct validity of the SC-IdFAI

Correlation coefficient *r*_*s*_ (*P* value) ^a^	C-IdFAI subscales				
	Overall Scale	Factor 1	Factor 2	Factor 3	Hypotheses
**FAAM**					
ADL	− 0.776 (< 0.001) ^b^	− 0.668 (< 0.001) ^b^	− 0.575 (< 0.001) ^b^	− 0.738 (< 0.001) ^b^	≥ Moderate, and better than SF-36 wih SC-IdFAI
Sport	− 0.775 (< 0.001) ^b^	− 0.644 (< 0.001) ^b^	− 0.636 (< 0.001) ^b^	− 0.669 (< 0.001) ^b^	
**SF-36**					
Physical Function	− 0.412 (< 0.001) ^b^	− 0.395 (< 0.001) ^c^	− 0.295 (0.001) ^c^	− 0.344 (< 0.001) ^c^	≥ Moderate, and worse than FAAM wih SC-IdFAI
Role-Physical	−0.551 (< 0.001) ^b^	− 0.511 (< 0.001) ^b^	− 0.405 (< 0.001) ^b^	− 0.424 (< 0.001) ^b^	
Bodily Pain	− 0.482 (< 0.001) ^b^	− 0.441 (< 0.001) ^b^	− 0.359 (< 0.001) ^c^	− 0.422 (< 0.001) ^b^	
General Health	−0.549 (< 0.001) ^b^	− 0.529 (< 0.001) ^b^	− 0.323 (< 0.001) ^c^	−0.511 (< 0.001) ^b^	
Vitality	−0.372 (< 0.001) ^b^	− 0.404 (< 0.001) ^c^	− 0.155 (0.075) ^b^	−0.278 (0.001) ^b^	≤ Poor, and worse than physical subscales of SF-36 and SC-IdFAI
Social Function	−0.242 (0.005) ^b^	−0.173 (0.047) ^b^	− 0.230 (0.008) ^b^	−0.107 (0.223) ^b^	
Role-Emotional	0.035 (0.694) ^b^	0.066 (0.455) ^b^	−0.048 (0.586) ^b^	0.050 (0.568) ^b^	
Mental Health	−0.137 (0.119) ^b^	−0.116 (0.186) ^b^	− 0.083 (0.345) ^b^	−0.141 (0.108) ^b^	

^a^ Calculated by the Spearman’s correlation coefficient (*r*_*s*_) of the overall scale and subscales of the SC-IdFAI with FAAM and SF-36

^b^ Consistent with the Hypothesis

^c^ Inconsistent with the Hypothesis

*SC-IdFAI* Simplified Chinese version of the Identification of Functional Ankle Instability questionnaire; *FAAM* Foot and ankle ability measure; *ADL* Activity of daily living; *SF-36* Short-Form 36

### Responsiveness

Finally, the results of the 86 patients with CAI who completed the SC-IdFAI questionnaire before and after physiotherapy (9-week interval) were used, and the ES and SRM were calculated to assess the responsiveness. As shown in Table 3, the overall ES and SRM of the SC-IdFAI were 1.123 and 1.554, respectively. The ES and SRM of the subscales, except for subscale factor 1, were also higher than 0.8. These findings demonstrated that the SC-IdFAI questionnaire had good responsiveness.

## Discussion

PROMs are essential tools for clinical studies. Investigators can use these scales to quantitatively assess the function and status of patients and compare the results of the scales with those reported in other similar studies, which can be used to guide the increasing number of multicenter clinical studies [6]. Currently, in China, several clinical studies are conducted and published every year, which are associated with not only large patient populations but also attention and support from the government [45]. Therefore, validated PROMs are urgently needed in China, as they can not only help a large number of patients receive more accurate diagnoses and appropriate treatments but also support the increasing number of clinical studies in China. Currently, however, no Chinese version of the disease-specific scale is available in China for a large number of patients with CAI. The IdFAI questionnaire has been acknowledged to be the most accurate and simple specific scale for assessing CAI, as it is based on the CAIT and AII. The IdFAI questionnaire has already been translated into four languages and has shown good reliability and validity in several independent studies [11, 13, 14, 19, 20]. Therefore, translating the IdFAI questionnaire into the Chinese language is valuable.

Before the results are discussed, the limitations of this study deserve our attention. First, China’s population may not be fully represented because the sample size is limited. However, according to an epidemiological study on CAI conducted with a large sample size (829,791 CAI patients) and published by Oded Hershkovich et al., the prevalence of CAI is significantly higher in males than in females, while the number of male patients in this study was also higher than that of females (male/female ratio = 86/46). In addition, the authors found that the highest incidence of CAI occurred in individuals aged between 15 and 24 years, while the proportion of young patients under 30 years old in this study was 78.8%. Therefore, it can be stated that the population composition of the study basically conforms to the epidemiological trend [45]. Second, we translated the scale using simplified Chinese, as it is the official language in China. However, China is a multiethnic country, and many ethnic groups in China used their own languages. Hence, in the survey, ethnic cultural differences deserve attention. Finally, some participants were excluded, and some were lost to follow-up, but overall, the sample appeared to be adequately powered based on the results.

The Cronbach’s *α* value of the SC-IdFAI questionnaire was slightly higher than those of the Brazil (0.87), Japanese (0.87), and Korean versions (0.89) but slightly lower than those of the original (0.96) and Persian versions (0.95). However, Cronbach’s *α* values higher than 0.95 generally suggest the existence of redundant questions in the scale. Therefore, not all the higher Cronbach’s *α* values suggested a better scale [30]. The three subscales of the SC-IdFAI questionnaire also had acceptable or good internal consistency (Cronbach’s *α* = 0.725–0.882), which was generally in agreement with the results of other previous studies [11, 14, 19]. The findings also showed that omitting any of the questions would not evidently increase the Cronbach’s *α* value of the SC-IdFAI questionnaire (0.880–0.904). However, the findings for the Korean version showed that omitting question 8 increased the Cronbach’s *α* value of the IdFAI-K questionnaire [20]. The investigators speculated that the results could be affected by professional athletes being included as study participants, as question 8 was as follows: “Following a typical incident of your ankle rolling over, how soon does it return to normal?” Thus, the inclusion of professional athletes can substantially affect the responses to this question. In contrast, the participants in this study and other previous studies were all members of the general population and not professional athletes, which can lead to differences between the results in these studies and those of the Korean version. The three subscales of the SC-IdFAI questionnaire also had good or excellent test–retest reliability (ICC = 0.876–0.936), which was in agreement with the findings reported by previous studies. In addition, a 1-week interval was assumed to be appropriate for assessing test–retest reliability, as 1 week was sufficient for the patients to forget the exact answers they provided for the first survey but not sufficient for the functional status and daily lives of the patients to change evidently. In addition, some patients had to wait for the first physiotherapy session for 1 week. Therefore, they did not receive other treatments in this time interval (those who received other treatments were excluded after completing the second survey) to prevent related biases from affecting the results. The MDC (I) and MDC (G) values of the SC-IdFAI questionnaire were 3.73 and 0.32, respectively. Scores of the two scales that were higher than 3 suggested real differences in the ankle functions of the two subjects. The MDC and low values for measurement error indicated that small clinical changes can be detected not only at the population level but also at the individual level by the SC-IdFAI.

In this study, the mean score of the SC-IdFAI questionnaire was slightly higher than those of the other versions (14.3–20.38) [11, 19], suggesting that CAI was relatively more severe in the patients in this study. In addition, the ceiling effect of the SC-IdFAI was 1.52% rather than 0, as shown in other versions, but the percentage of the patients was far smaller than 15%, suggesting that very few patients had the maximum score on the scale. The three experts also confirmed that the items of the SC-IdFAI questionnaire were relevant for the construct to be measured and for patients with CAI. No missing questions were found in the recovered SC-IdFAI questionnaire. These findings and the good feedback from the patients suggested that the SC-IdFAI questionnaire had good content validity.

In the studies on the Korean version of the IdFAI questionnaire, the criterion validity of the scale was assessed [20]. However, according to the COnsensus-based Standards for the selection of health status Measurement Instruments (COSMIN) checklist, the “criterion validity” was defined as the degree to which the scores of a PROM instrument are an adequate reflection of a “gold standard” [36]. The COSMIN checklist is a consensus-based checklist used to evaluate the methodological quality of studies conducted on the measurement properties of health status measurement instruments based on an international Delphi study in 2010. The criterion used should be considered a reasonable “gold standard”, but the Delphi panel reached a consensus that no gold standards exist for PROM instruments [36]. Therefore, the criterion validity of the scale could not be assessed. The methods used in the Korean version were more likely for assessing the construct validity of the scale. In addition, the studies on other versions of the IdFAI questionnaire did not assess the construct validity of the scale using “hypotheses testing”, as recommended by the COSMIN checklist [11, 13, 14, 19, 20]. Such studies only calculated the correlation coefficient of the translated IdFAI questionnaire with other control scales. However, no clearly quantified indexes exist for the eligibility of the “construct validity” of the translated IdFAI. Therefore, it was speculated that proposing a series of hypotheses according to the COSMIN checklist first and then assessing whether the results were in agreement with the hypothesis can be performed to assess the construct validity of the scale, as this method had predefined indicators. In this study, the hypothesis testing method was used.

Responsiveness was not assessed in the original version or other versions of the scale [11, 13, 14, 19, 20], despite the importance of responsiveness; it is an important indicator reflecting whether a scale can be used in prospective clinical studies. In this study, the SC-IdFAI showed good responsiveness, which means that the SC-IdFAI can be sensitive to changes in the functional condition of patients after systemic physiotherapeutics.

## Conclusions

In summary, we have successfully translated the IdFAI into Chinese. After verification, the Chinese version was determined to easy to use and have good responsiveness, reliability and validity. Therefore, the results of this study suggest that the SC-IdFAI questionnaire can be used in future clinical studies and clinical practices to assess the functions of Chinese patients with CAI, thereby helping physicians and investigators better obtain required data.

## Data Availability

The data are contained within the manuscript and the datasets supporting the conclusion of this article are available from the corresponding author upon reasonable request.

## Abbreviations

IdFAI: The Identification of Functional Ankle Instability questionnaire
AII: Ankle Instability Instrument
CAIT: Cumberland Ankle Instability Tool
CAI: Chronic ankle instability
SF-36: Medical Outcomes Study Short-Form 36
FAAM: Foot and Ankle Ability Measure
SEM: Standard error of measurement
MDC: Minimal detectable change
PROMs: Patient reported outcome measures
WOSI: Western Ontario Shoulder Instability Index
NATA: National Athletic Trainers’ Association
ES: Effect size
ICC: Intra-class correlation coefficient
SRM: Standardized response mean
